# Physiologic Implications of Reactive Oxygen Species Production by Mitochondrial Complex I Reverse Electron Transport

**DOI:** 10.3390/antiox8080285

**Published:** 2019-08-06

**Authors:** John O. Onukwufor, Brandon J. Berry, Andrew P. Wojtovich

**Affiliations:** 1 Department of Anesthesiology and Perioperative Medicine, University of Rochester Medical Center, Rochester, NY 14642, USA; 2 Department of Pharmacology and Physiology, University of Rochester Medical Center, Rochester, NY 14642, USA

**Keywords:** reactive oxygen species, mitochondrial complex I, reverse electron transport, superoxide, hydrogen peroxide, ischemia reperfusion injury, oxidative damage

## Abstract

Mitochondrial reactive oxygen species (ROS) can be either detrimental or beneficial depending on the amount, duration, and location of their production. Mitochondrial complex I is a component of the electron transport chain and transfers electrons from NADH to ubiquinone. Complex I is also a source of ROS production. Under certain thermodynamic conditions, electron transfer can reverse direction and reduce oxygen at complex I to generate ROS. Conditions that favor this reverse electron transport (RET) include highly reduced ubiquinone pools, high mitochondrial membrane potential, and accumulated metabolic substrates. Historically, complex I RET was associated with pathological conditions, causing oxidative stress. However, recent evidence suggests that ROS generation by complex I RET contributes to signaling events in cells and organisms. Collectively, these studies demonstrate that the impact of complex I RET, either beneficial or detrimental, can be determined by the timing and quantity of ROS production. In this article we review the role of site-specific ROS production at complex I in the contexts of pathology and physiologic signaling.

## 1. Introduction

Mitochondria are a major source of reactive oxygen species (ROS) production in cells. ROS can be produced during cellular respiration [1,2], where electrons pass directly onto oxygen instead of the next electron carrier in the electron transport chain (ETC) [2,3,4,5]. There are many antioxidant systems that detoxify ROS [6]. Under certain conditions, however, such as damage to the ETC or during ischemia reperfusion injury, the amount of ROS produced overwhelms the cellular defense systems resulting in oxidative stress [2,3,7]. For many years ROS have been associated only with oxidative stress, however, it is now accepted that ROS play a role in cellular signaling as well [7,8,9,10,11,12]. Determining what factors make ROS beneficial or detrimental is an active area of research [13]. Much like the second messenger calcium, the effect of ROS can be determined by many factors including how much, when, and where ROS are produced. There are many sites of ROS generation in mitochondria [1,2,14,15,16] each of which is differentially sensitive to cellular conditions [17]. However, our understanding of how site-specific ROS production contributes to physiology remains limited. Here we will focus on ROS generated at mitochondrial complex I of the ETC. Complex I can generate ROS through reverse electron transfer (RET) under a variety of metabolic conditions, many of which are associated with pathology [2,12,18,19]. However, recent studies suggest that complex I ROS generation by RET can have physiologic roles [12,20,21,22,23,24].

## 2. Complex I ROS Generation

### 2.1. Complex I Function and ROS Production

**Figure 1:**
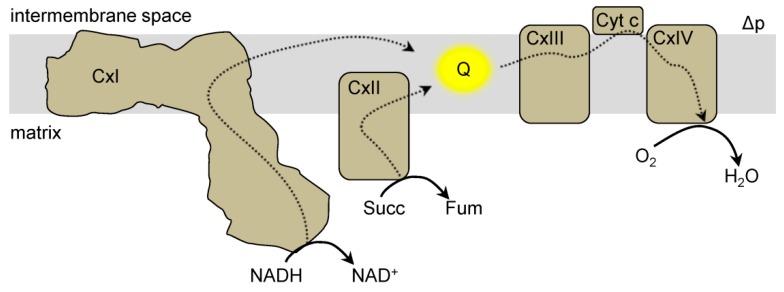
Mitochondrial electron transport chain. The electron transport chain (ETC) is located in the mitochondrial inner membrane. Electrons (dashed lines) from reducing equivalents (e.g., NADH) enter the ETC at complex I (CxI) or complex II (CxII) and reduce the ubiquinone pool (Q, yellow). Electrons are then transferred from QH_2_ to complex III (CxIII), to cytochrome c (Cyt c) and ultimately onto oxygen (O_2_) at complex IV (CxIV). During this process protons are pumped (not shown) to generate a protonmotive force (Δp) which is comprised of a membrane potential and a proton concentration gradient.

Mitochondrial complex I (NADH:ubiquinone oxidoreductase) is a large multimeric protein complex and a member of the ETC [25,26,27,28]. Complex I is comprised of a hydrophobic section located in the inner membrane and a hydrophilic section in the matrix (Figure 1). Complex I transfers two electrons from NADH to ubiquinone (Q) and pumps protons into the intermembrane space [27,29]. The hydrophilic section of complex I catalyzes the oxidation of NADH at the flavin mononucleotide (FMN) containing subunit. Electrons are then passed through a series of iron-sulfur clusters to reduce Q to ubiquinol (QH_2_) at the Q binding site [26,27]. QH_2_ can transfer electrons to complex III, which are eventually passed onto oxygen at complex IV to form water [27,29]. During this process, an electron can reduce oxygen to generate superoxide (O_2_•^−^) instead of continuing down the ETC. O_2_•^−^ can be converted to hydrogen peroxide (H_2_O_2_) through spontaneous dismutation or enzymatically via superoxide dismutase (SOD) [2]. There are many sites of ROS production in mitochondria, and complex I can generate ROS either through forward electron transfer (NADH to Q) or reverse electron transfer (QH_2_ to NAD^+^) [30]. Sites of O_2_•^−^ generation within complex I include the FMN group, iron-sulfur clusters, and the Q binding site [31,32,33,34,35]. The FMN and Q sites are relatively accessible to the mitochondrial matrix. These locations have high probabilities of electrons leaking from the ETC and reducing oxygen to O_2_•^−^ [26,27]. Additionally, each ROS production site within complex I has a different sensitivity to oxygen concentration, such that changes in the local oxygen levels can affect the site’s contribution to ROS production [17].

### 2.2. Protonmotive Force (Δp) and Complex I ROS

The mitochondrial protonmotive force (Δp) regulates RET ROS production. The Δp is composed of two potential energies—a proton concentration gradient (ΔpH) and a charge separation known as the membrane potential (Δψ_m_) [35]. Under normal conditions, the Δψ_m_ is the major component of the Δp [2]. During an energy demand mitochondria use the Δp to produce ATP, and ROS generation is low [36]. Likewise, protonophores, such as FCCP or DNP, dissipate the Δp and decrease complex I ROS production [35,37,38]. At rest conditions, when the Δp is relatively high, ROS production is higher [35]. Therefore, a high Δp is associated with a large amount of complex I ROS generation, and that decreasing the Δp will inhibit complex I ROS production. However, it is unknown whether ΔpH and Δψ_m_ have different contributions to ROS production, or if total Δp is all that matters.

To determine the contribution of ΔpH and Δψ_m_ to complex I ROS, ion transporters were used to experimentally test each parameter. For example, nigericin lowers ΔpH and increases Δψ_m_ to maintain Δp [35]. Under these conditions, complex I ROS decreased suggesting that complex I ROS is driven by a high ΔpH [35]. Similarly, phosphate carrier activity decreased the ΔpH and ROS production [35]. Recently, however, using both isolated heart and brain mitochondria, increasing the ΔpH and decreasing the Δψ_m_ simultaneously using a potassium ionophore was concomitant with decreased ROS production [38]. Then using nigericin to perform the reverse experiment (decrease the ΔpH and increase the Δψ_m_), increased ROS production was observed. Importantly, these changes were associated with changing pH levels [38], suggesting that the absolute pH, rather than the ΔpH across the inner membrane is responsible for the high complex I ROS production [38].

### 2.3. Redox Ratios and Complex I ROS

The redox statuses of the QH_2_/Q and NADH/NAD^+^ ratios are also major determinants of the amount of ROS produced at complex I. For example, when mitochondria are making ATP the NADH/NAD^+^ ratio is low and ROS production is also low [2]. Conversely, a high NADH/NAD^+^ or QH_2_/Q ratio is associated with a high level of ROS production [2,39,40]. In conclusion, there are various factors that control the production of ROS and many of these factors are interconnected. For example, changes in the Δp can change redox ratios. Therefore, altering one of these factors in vivo can lead to metabolic changes and introduce confounding factors.

## 3. Mitochondrial Complex I Reverse Electron Transfer (RET)

**Figure 2:**
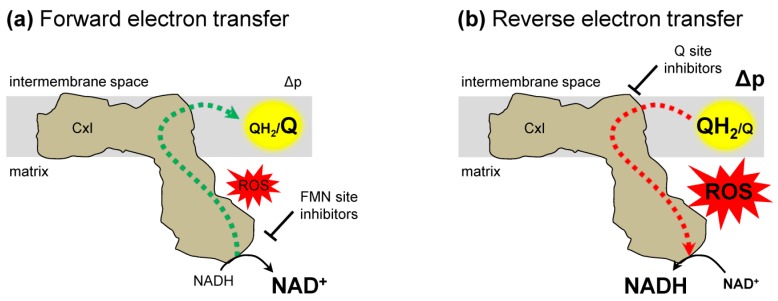
Complex I electron transfer. (**a**) Forward electron transfer. Electrons (dashed lines) are transferred from NADH to ubiquinone (Q) resulting in ubiquinol (QH_2_). These conditions can generate reactive oxygen species (ROS). The addition of FMN site inhibitors prevent, while Q binding site inhibitors enhance complex I forward electron transfer generated ROS. (**b**) Reverse electron transfer (RET). Electrons from ubiquinol (QH_2_) are transferred to NAD^+^ and generate ROS. Both Q binding site and FMN binding site inhibitors suppress RET generated ROS. Δp, protonmotive force; FMN, flavin mononucleotide.

The electron flow in complex I is described in two modes—forward and reverse electron transfer (Figure 2). Under conditions of normal respiration, forward electron transfer (Figure 2a) is the energetically favored complex I-mediated transfer of electrons from NADH to Q. Reverse electron transfer (RET) (Figure 2b) is the backward transfer of electrons from QH_2_ to NAD^+^ [2,12]. Under normal conditions, RET is energetically uphill and requires a high Δp and QH_2_/Q ratio [30,41,42]. RET is not tissue or organ specific, as it occurs in mitochondria from different tissues [17,33,35,39,42,43]. In addition, though RET was first observed under conditions of succinate respiration in isolated mitochondria, it is not linked solely to this condition. Mitochondria fueled with fatty acids or α-glycerophosphate to reduce Q can undergo RET [1,21], suggesting that RET can be influenced by multiple metabolic pathways.

RET is distinguished from other mechanisms of complex I ROS generation through the use of inhibitors. RET generation of ROS is inhibited by Q binding site inhibitors, such as rotenone [35,42,44]. The specific site responsible for RET ROS production within complex I (e.g., FMN site or Q binding site) is debated [1,2,35,44,45], but all of the likely sites of RET ROS production are located in the mitochondrial matrix [44]. However, current O_2_•^−^ detection methods lack spatial resolution to precisely distinguish matrix vs. IMS ROS production [26,27]. For example, biosensors, which can be targeted to the matrix or intermembrane space, respond to the membrane permeable H_2_O_2_, not O_2_•^−^. Mitochondrial ROS plays a role in cell signaling yet, until recently, the importance of RET ROS in modulating physiologic processes has been overlooked. Since the conditions required for RET (high Δp and QH_2_/Q ratio) are not often present in physiologic circumstances, RET has been largely associated with pathologic conditions [41,42,46]. New studies are demonstrating a role for RET generated ROS in physiologic processes [1,2,12,20,21,33,47].

## 4. Detecting and Modulating RET ROS Generation

### 4.1. Detecting RET

RET generates O_2_•^−^, which can dismutate to H_2_O_2_, both of which are commonly measured as an output of RET [2]. Extensive analysis of ROS detection methods are reviewed elsewhere [48,49]. Here, we will focus on detection methods that relate to measurements of physiologic RET. O_2_•^−^ is commonly measured using dihydroethidium (DHE) or mitoSOX (i.e. mitochondria-targeted DHE) [50,51,52]. DHE can be oxidized through many mechanisms to yield a range of oxidation products. Of the two fluorescent DHE oxidation products, ethidium (E^+^) and 2-hydroxyethidium (2-OHE^+^), only 2-OHE^+^ is a selective marker of O_2_•^−^ [53]. The spectral overlap of E^+^ and 2-OHE^+^ and their propensity to intercalate DNA limit the interpretation of fluorescent measurements of O_2_•^−^ as a readout for RET [53]. To accurately detect O_2_•^−^ using DHE, the oxidation products should be separated and quantified using HPLC [50,51,52,54]. Recent advances minimized concerns by modifying structural features of mitoSOX to create mitoNeoD [55], which may limit the confounding factors associated with DHE fluorescence readouts as a measure of RET generated O_2_•^−^ in vivo [55].

An alternative way to assess complex I RET ROS is to measure H_2_O_2_ using Amplex Red, which in the presence of horseradish peroxidase will react with H_2_O_2_ to give the fluorescent product resorufin [2]. While the use of Amplex Red is well established, studies are generally limited to isolated mitochondria. In vivo studies use ROS biosensors targeted to specific cellular regions or compartments to measure H_2_O_2_ [56,57,58,59,60]. The oxidation of biosensors such as HyPer or redox-sensitive GFP-based probes results in a reversible change in fluorescent signal [61,62]. Like most fluorescent proteins, however, biosensors can be sensitive to pH and require appropriate control experiments [61]. For example, the pH gradient across the mitochondrial inner membrane can confound interpretation of ROS readouts when comparing a biosensor signal in the mitochondrial matrix to the intermembrane space or cytosol because of pH differences. Advances in biosensors are increasing the dynamic range and sensitivity to H_2_O_2_ while limiting the influence of pH [61].

### 4.2. Modulating RET 

**Figure 3:**
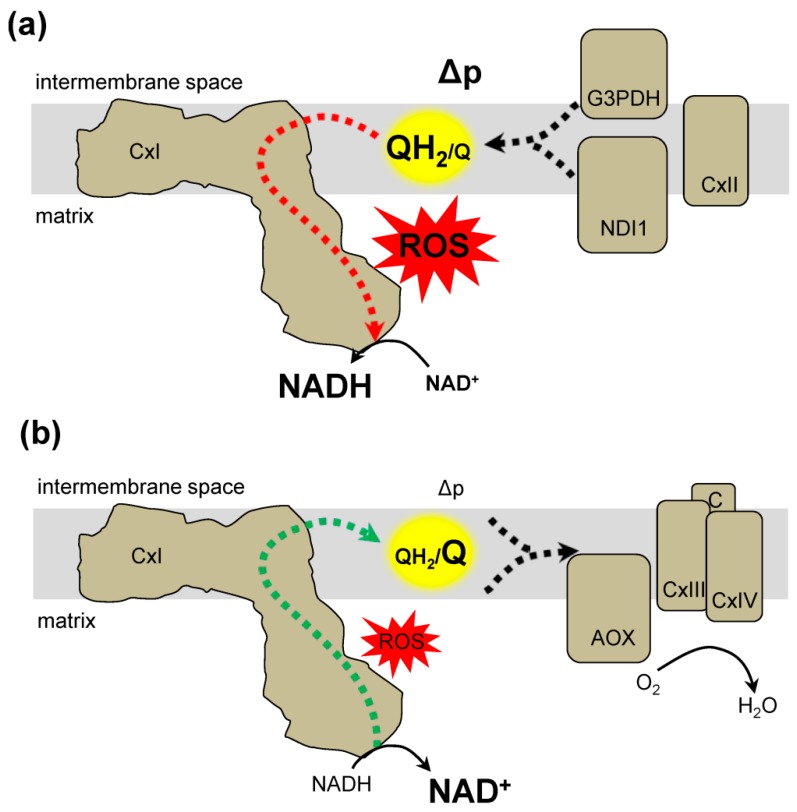
Genetic approaches to alter the QH_2_/Q ratio and modify complex I reverse electron transfer. (**a**) The expression of NDI1 in the mitochondria can result in the reduction of the Q pool. NDI1 transfers electrons from NADH to Q resulting in QH_2_. The resulting conditions favor reverse electron transfer generation of ROS. Other proteins that can increase the QH_2_/Q ratio include complex II (CxII) or glycerol-3-phosphate dehydrogenase (G3PDH). (**b**) Expression of alternative oxidase (AOX) decreases the QH_2_/Q ratio. AOX transfers from QH_2_ to O_2_ to produce water (H_2_O). Together the activity of complex III (CxIII), cytochrome c (C), and complex IV (CxIV) also decrease the QH_2_/Q ratio resulting in conditions that do not favor RET formation of ROS.

In addition to detecting RET, ROS production can be modulated both pharmacologically with Q site inhibitors, and genetically through expression of exogenous electron transfer systems. RET ROS production can be differentiated from forward electron transfer by using rotenone. RET O_2_•^−^ production is inhibited by rotenone, where forward electron transfer ROS production is not. Another way to probe RET is by manipulating the QH_2_/Q ratio to influence the rate of complex I ROS production [2]. This approach is implemented by using exogenous enzymes to mimic mitochondrial electron transfer. Expression of *Ciona intestinalis* alternative oxidase (AOX) and the yeast NADH dehydrogenase (NDI1) is a novel approach to control the QH_2_/Q ratio (Figure 3) [20,21]. AOX is a cyanide-insensitive oxidase that transfers electrons from QH_2_ to oxygen (Figure 3b). The expression of AOX can decrease the QH_2_/Q ratio [63]. Characterization of AOX in plants suggests that it is active only once the QH_2_/Q ratio reaches a threshold, suggesting a regulatory switch [64]. NDI1 is a rotenone-insensitive NADH dehydrogenase that transfers electrons from NADH to Q, resulting in a high QH_2_/Q ratio (Figure 3a) [65]. Together AOX and NDI1 can bypass components of the ETC to directly alter the QH_2_/Q ratio. However, there are many electron entry points to the Q pool, such as complex II, dihydroorotate dehydrogenase, electron transfer flavoprotein-ubiquinone oxidoreductase, and glycerol-3-phosphate dehydrogenase. AOX expression does not affect development in *Drosophila melanogaster* and suggests that AOX expression is not detrimental [63,66]. However, it is unclear how the AOX-mediated changes in the QH_2_/Q ratio can impact metabolism and subsequently, ROS production at other mitochondrial sites. Recently, AOX expression was shown to decrease male fertility in *Drosophila melanogaster* [67]. While the role of ROS in mediating fertility is unclear, it demonstrates that AOX can influence physiologic processes. Additionally, AOX activity will consume oxygen and can effect ROS production at the substrate level, since altering local oxygen concentrations can impact ROS production rates [68]. Overall, multiple approaches and careful interpretation are warranted to determine the role of complex I RET generated ROS.

## 5. RET Generated ROS in Pathology

**Figure 4:**
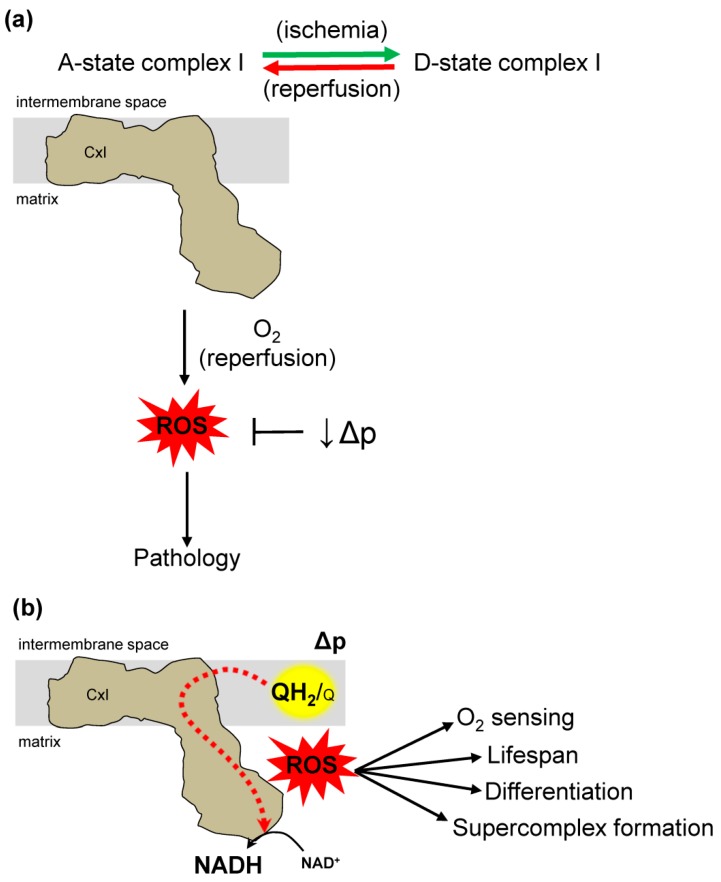
Dual role of complex I reverse electron transfer in pathology and physiology. (**a**) Complex I (CxI) reverse electron transfer (RET) in pathology. Factors that affect RET in ischemia reperfusion (IR) injury. During ischemia, succinate accumulation drives RET at complex II forcing electrons to complex I, where ROS is subsequently overproduced. Complex I has two states, active (A) and dormant (D). Ischemia drives an A-form that transition to D-form. During reperfusion D-form transition back to A-form generating reactive oxygen species (ROS) that causes IR injury. The mitochondrial protonmotive force (Δp) is a main driving force for RET. Therefore, decreasing the Δp removes a main driver of RET and ROS production at reperfusion. (**b**) Complex I reverse electron transfer contributes to diverse physiologic processes. The site-specific ROS production has a role in oxygen (O_2_) sensing, lifespan extension, cell differentiation, and supercomplex formation.

Most of what is known about RET was discovered in models of ischemia reperfusion (IR) injury. The conditions for RET described herein occur in many pathologic situations, but we will focus IR injury. Loss of blood flow to tissue (ischemia) and subsequent reestablishment (reperfusion) results in IR injury. In mitochondria, depleted oxygen during ischemia combined with its rapid restoration at reperfusion cause oxidative damage to tissue [69]. This damage can occur from ROS generated in several places in mitochondria [70]. At complex I, metabolic substrates that accumulate during ischemia [18] are rapidly oxidized at reperfusion, causing damage. Specifically, accumulated succinate drives RET and ROS production, implicating complex I ROS in the oxidative damage from IR injury (Figure 4a) [19].

Complex I has enzymatically active (A) and dormant (D) states under different conditions [71]. The A state has catalytic NADH/Q oxidoreductase activity, however, the D state is inhibited and more susceptible to oxidative modification in vitro [72,73]. During ischemia there is a reversible transition from the A to the D state. This transition is hypothesized to be a physiologic mechanism to limit ROS production at reperfusion, as the D state makes less ROS by RET than the A state [74]. Oxidative damage at reperfusion is driven by RET succinate oxidation at functional complex I in the A state [30]. Ischemic transition of complex I to the D state could be a preparation for reperfusion, serving to inhibit complex I activity and RET (Figure 4a). In depth characterization of complex I A/D transition could yield targeted therapies to prevent damage. Targeting specific sites of complex I ROS formation could also yield targeted therapy.

During RET, one of the ROS production sites in complex I is the Q binding site (IQ) [75]. There are many small molecules called suppressors of complex I site I(Q) electron leak (S1QELs), that inhibit complex I at this site, preventing ROS production and protecting against IR injury [70,76,77]. The onset of IR events is unpredictable, however. Using drugs to protect against IR injury would be most clinically useful administered at or after reperfusion, when it is clear intervention is needed. In support of this notion, metformin, an FDA approved diabetes drug, can inhibit complex I at reperfusion and limit IR injury in animal and cell models [78]. Similarly, genetic knockout of a complex I subunit results in altered ROS levels and decreased susceptibility to IR injury [79]. These results suggest diminished complex I activity, and resulting diminished RET activity at reperfusion, is protective against IR. These data are in line with the proposed beneficial role of D state complex I preventing oxidative damage at reperfusion. While preemptively inhibiting complex I ROS production before an ischemic event protects against IR injury, the inhibition could impair beneficial oxidative signaling, as discussed in the following section. The dual roles of ROS damage and signaling may be the reason for the failure of global antioxidant treatments for disease [80,81]. Again, targeted approaches to decrease damaging ROS while preserving signaling ROS will be necessary for the successful development of redox medicine. This approach requires in-depth understanding of the molecular mechanisms of RET and complex I function.

Changes in the Δp can also alter RET, where decreased potential results in less ROS production (Figure 4a) [35]. Decreasing both components of the Δp with small-molecule protonophores like FCCP is protective in many models of IR injury [82,83,84]. The molecular details of these and similar results are unclear but represent a promising targeted approach to understand mechanisms of pathology.

The research summarized here demonstrates how RET and complex I ROS production contribute to IR injury, and shows that clarifying molecular mechanisms will be essential for clinical application. The pathologic events in and around mitochondria during IR are interrelated and coupled to cellular metabolism [36]. Thus, careful experimental approaches will isolate RET to discern mechanisms in pathology.

## 6. RET Generated ROS in Physiology

Although RET is involved in pathology, it may have roles in well-coordinated physiological processes that cause cellular adaption (Figure 4b). For example, RET generated ROS is implicated in detecting cellular oxygen levels, cellular differentiation, lifespan extension, and ETC rearrangement [12,20,21,22,23,24].

The ability to sense limited oxygen and initiate a rapid adaptive response is key to cellular survival under low oxygen tensions. One way that an organism senses low oxygen is through the carotid body [11,22,85]. The carotid body is a group of cells in the carotid artery responsible for oxygen sensing that contain oxygen-sensitive K^+^ channels. During hypoxia, K^+^ channels are inhibited, leading to cellular depolarization and the release of neurotransmitters, resulting in hyperventilatory activity [22]. Hyperventilation (rapid breathing) is an acute way the body increases oxygen supply to tissues [86]. The precise mechanism through which the carotid body senses low oxygen and activates a response is still unfolding [22], however, RET generated ROS is involved [22,23]. This is based on the observation that high carotid body activity following low oxygen tension corresponded with elevated ROS measured by mitoSOX fluorescence in carotid body cells [22]. In addition to mitoSOX, a redox-sensitive fluorescent protein sensor (roGFP) was targeted to the mitochondrial intermembrane space and to the cytosol to measure ROS levels in mice exposed to hypoxia [22]. The levels of H_2_O_2_ were high in the intermembrane space and low in the cytosol. The changes in ROS levels in carotid body cells were sensitive to the loss of *Ndufs2*, which encodes the complex I ubiquinone binding site, suggesting that ROS originates from complex I [22]. These results highlight the importance of site-specific measurement of ROS [23]. Given that ETC inhibitors other than rotenone can influence carotid body cell oxygen sensing [87], and that other sources of ROS can mediate responses to hypoxia [88], understanding the site-specific nature of complex I ROS in the signaling process is essential. In support of this notion, a recent paper reinforced the oxygen sensing role of *Ndufs2* [89].

RET has also been implicated in muscle differentiation [24]. ROS produced at complex I stimulated muscle differentiation in H9c2 rat cardiac myoblasts [24]. The increased mitochondrial O_2_•^−^ was measured by mitoSOX fluorescence and was associated with an increase in SOD expression and H_2_O_2_ diffusion to the cytosol, where it stimulated differentiation [24]. Mitochondria-targeted O_2_•^−^ scavengers, mitoquinone (mitoQ) and mitoTempol suppressed morphological changes, major histocompatibility complex (MHC) expression in myotube formation, muscle creatine kinase (MCK) promoter activity and H_2_O_2_ production, confirming the role of ROS in muscle differentiation. Moreover, the expression of mitochondria-targeted catalase (mCAT) prevented differentiation, implicating the mitochondrial matrix ROS production [24]. Three subunits of complex I were upregulated during muscle differentiation, suggesting that complex I plays a role in O_2_•^−^ formation [24]. In agreement with these studies, mitochondrial ROS and muscle differentiation were sensitive to rotenone and the knockdown of the complex I subunits *Ndufaf1* and *Ndufs3* [24]. In addition, a single bout of exercise resulted in increased mitophagy and mitochondrial function in both heart and skeletal muscle [90]. These increases were in part due to the increase in mitochondrial H_2_O_2_ measured using Amplex Red [90]. Similarly, dietary lutein, a carotenoid that aids vision, enhanced the differentiation of SH-SY5Y cells through phosphoinositide-3-kinase signaling, which is triggered by mitochondrial H_2_O_2_ [91]. These results are in line with conditions that favor RET, but a direct role of complex I ROS is unclear.

RET generated ROS also mediates lifespan extension, as observed in flies [20]. The role of RET in lifespan extension was investigated by modulating the redox state of the QH_2_/Q ratio using NDI1 and AOX [20,92,93]. The expression of NDI1 altered the steady-state ratio of NADH/NAD^+^, reduced Q, and extended lifespan [92]. In a pharmacologic approach, rotenone (a Q binding site inhibitor) treatment or knockdown of complex I did not alter the beneficial effects of NDI1 [92], suggesting that NDI1 compensates for endogenous complex I activity in flies [92]. Interestingly, NDI1 does not compensate for lost complex I activity in *C. elegans* [94,95], suggesting that QH_2_/Q ratio can be differentially regulated in various organisms. In support of this notion is the observation that AOX inhibited the lifespan extension by NDI1 [20]. This indicates that QH_2_ is at the center of the lifespan extension. In line with this, feeding flies rotenone reduced ROS production in NDI1 mutant flies but not in wildtype flies [20]. Similar results were observed using protonophores to dissipate the Δp. This indicates the involvement of complex I RET as the source of NDI1 mediated ROS production that extends lifespan in flies. If the mechanisms are conserved in mammals, manipulation of the QH_2_/Q ratio provides a therapeutic target for the extension lifespan, or for treatment of aging and age-related disease such as IR injury and diabetes [20].

RET can contribute to physiology through changes at the organelle level. During an energy demand the ETC complexes can arrange into supercomplexes, which include groupings of complexes I, III, and IV of the ETC, and their formation is associated with efficient respiratory activity [96]. RET is implicated in the formation of supercomplexes following changes in fuel source, oxygen tension, or stress [21,96,97]. When the ETC complexes are fueled through glycolysis, electrons from NADH can increase the QH_2_/Q ratio via complex I. These conditions favor a supercomplex where complex I is associated with complex III [12,21]. β-oxidation can further reduce the Q pool through the added contribution of FAD-dependent enzymes. The resulting increased QH_2_/Q ratio is associated with high levels of ROS production and disassociated supercomplexes. Recent work demonstrated that the high QH_2_/Q ratio during β-oxidation leads to RET produced ROS (Figure 3a) and the localized RET leads to changes in supercomplex formation. The localized ROS were shown to oxidize complex I, resulting in the degradation and dissociation of complex I from the supercomplex [21]. Without a privileged location between complexes I and III, the dissociated complex III is hypothesized to favor electron flux through FAD-dependent sources and limit overall ROS production [21].

Overall, RET ROS production has defined roles in physiology. Understanding the molecular details of RET could provide new mechanisms of physiologic regulation.

## 7. Conclusions and Future Directions

Overall, where ROS are generated can greatly impact the cellular response. As such, ROS are involved in heterogeneous cell processes, ranging from signaling events to death. Our understanding of how RET generated ROS produces specific signals is limited. For example, it is unclear how RET ROS increases lifespan, and the signaling mechanisms involved are not fully elucidated. Further, it is unknown how each of these processes are selective for RET derived ROS. It is also unclear which sites of ROS production within complex I impact physiologic outputs. Answering these questions will help uncover the beneficial effects of site-specific ROS production. Careful experimental design and methods that rigorously measure site-specific RET ROS would increase our knowledge of physiologic RET. Methods of ROS detection are imperfect and are often used incorrectly, confounding interpretations. Focusing on defined molecular outputs of ROS rather than imperfect bulk measurements will advance the field in a complementary way. For example, measuring a specific ROS post-translational modification may be more informative than observing increased or decreased ROS under experimental conditions. This approach is important given the nuances of known microdomain effects [98,99]. The research described here highlights the importance of understanding molecular mechanisms of site-specific ROS production in mitochondria, and serves as a starting point to develop new methods to probe the many facets of ROS physiology.
